# The role of kaempferol in gynaecological malignancies: progress and perspectives

**DOI:** 10.3389/fphar.2023.1310416

**Published:** 2023-12-04

**Authors:** Xijun Ma, Xiaoyu Zhang, Xuan Wang, Congan Wang, Yuning Ma

**Affiliations:** ^1^ Neck-Shoulder and Lumbocrural Pain Hospital of Shandong First Medical University, Jinan, China; ^2^ School of Chinese Medicine, Shandong University of Traditional Chinese Medicine, Jinan, China; ^3^ Key Laboratory of New Material Research Institute, Institute of Pharmacy, Shandong University of Traditional Chinese Medicine, Jinan, China

**Keywords:** kaempferol, gynecologic malignant tumor, mechanism, pathway, cancer

## Abstract

Kaempferol, a flavonoid derived from various herbs such as cocoyam, propolis, and grapefruit, has garnered interest due to its numerous pharmacological benefits, including anti-inflammatory, antioxidant, and anti-diabetic properties. Kaempferol has been shown to possess notable anti-tumour bioactivity, indicating potential for treating gynaecological malignancies. To date, numerous studies have demonstrated the potential of kaempferol to induce tumour cell apoptosis, inhibit proliferation, and prevent metastasis and invasion in several gynaecological malignancies, including breast, ovarian and endometrial cancers. However, there is currently insufficient research investigating the efficacy of kaempferol for the treatment of gynaecological malignancies, and a lack of systematic review of its mechanism of action. Therefore, this review is founded on a literature analysis of the anticancer effects of kaempferol on gynaecological malignancies. The goal is to provide valuable reference material for scientific researchers and medical practitioners.

## 1 Introduction

Gynaecological malignancies are one of the leading causes of morbidity and mortality in women worldwide, seriously affecting their quality of life and health. The development of clinical tools, such as preventative vaccinations, early screening, surgical interventions, radiotherapy, and chemotherapy, has significantly advanced the prevention and treatment of these cancers (Schneider et al., 2020; Marcolin et al., 2023). However, in the present medical setting, unknown mechanisms, platinum resistance and complications in early diagnosis impede the prevention and treatment of gynaecological malignancies. Breast cancer incidence continues to rise each year (Britt et al., 2020), while ovarian cancer still lacks robust biomarkers and early detection methods (Zhang et al., 2022). Additionally, cervical (Chargari et al., 2022) and endometrial cancers (Katagiri et al., 2023) continue to grapple with drug resistance, relapse, and unfavourable prognosis. Hence, it is imperative to unravel the pathogenesis and drug resistance mechanisms of gynaecological malignancies and explore efficacious intervention targets and biomarkers for attaining optimal outcomes in the prevention and management of gynaecological malignancies.

Flavonoids are natural polyphenolic compounds that occur abundantly in fruits and vegetables commonly consumed by humans daily (Serafini et al., 2010; Sun et al., 2022; Chanu et al., 2023). They possess various biological activities, such as antioxidant, anticancer, and anti-inflammatory effects, and have been the subject of extensive research both domestically and internationally. Kaempferol is a flavonoid compound present in a diverse range of natural plant sources, including fruits, vegetables, and Chinese herbs (Periferakis et al., 2022). Recent research has indicated that kaempferol exerts numerous nutritional and health benefits, including anti-oxidant (Chagas et al., 2022), anti-inflammatory (Alam et al., 2020), anti-cancer (Akter et al., 2022), and treatment effects for conditions such as diabetes mellitus (Yang et al., 2022), atherosclerosis (Chen et al., 2022), and osteoporosis (Liu et al., 2021). Furthermore, kaempferol has demonstrated a neuroprotective effect (Chang et al., 2022), as well as beneficial effects for the liver (Xiao et al., 2022) and myocardium (Kamisah et al., 2023).

Kaempferol holds significant potential as a health food and medicine, with a broad market perspective. The pharmacological effects of kaempferol are currently under study, with the aim of further exploring its anti-tumour effects on gynaecological malignancies. The investigation of its anti-tumour mechanism is also a focus of scientific researchers’ attention. In recent years, advancements in bioinformatics, network pharmacology, and molecular docking technology have yielded dependable methods for predicting how kaempferol affects gynecological malignancies. Numerous researchers have utilized data mining to identify the potential targets of kaempferol’s impact on gynecological malignant tumours. Additionally, they utilized network pharmacology to investigate kaempferol’s effect on these tumours and predicted its outcomes through molecular docking technology. Using molecular docking technology, the binding sites and binding ability of kaempferol with anti-gynaecological malignant tumour targets were predicted. The predictions were tested in experiments to elucidate the mechanism underlying kaempferol’s action against gynaecological malignant tumours. Considering the current research situation, kaempferol displays noteworthy anti-tumour effects on gynaecological malignancies and potential for application due to its diverse mechanisms. However, current studies on kaempferol in gynaecological malignancies have certain limitations that need to be addressed. Accordingly, this review aims to summarise kaempferol’s mechanism in gynaecological malignancies to provide references for its therapeutic applications.

## 2 Structure and origin of kaempferol

Kaempferol, belonging to the flavonoids, is also referred to as kaempferol-3, kaempferol flavonol, and thymoquinone III. Its molecular structure formula is C15H10O6, with a relative molecular weight of 286.23 (Calderón-Montaño et al., 2011). The pure product of its monomer is a yellow crystalline powder with a melting point of 276°C–278°C. It is slightly soluble in water but can be dissolved in hot ethanol, ether, and alkali. The hydrophobicity of kaempferol is determined by its diphenylpropane structure. Its antioxidant activity is enhanced by hydroxyl radicals that can combine with the hydroxyl groups of C3, C5, C7 and C4′ (Calderón-Montaño et al., 2011). It is mainly obtained from the rhizome of Kaempferia galanga, which belongs to the Zingiberaceae family, and can be found abundantly in various fruits, vegetables and beverages (Imran et al., 2019). Kaempferol in its pure form has been extracted from various green plants including tea, cocoyam, witch hazel, propolis, and grapefruit.

## 3 Mechanisms of kaempferol against gynaecological malignancies

Numerous studies have demonstrated that long-term consumption of kaempferol, which is the most prevalent flavonoid, reduces the risk of cancer. Kaempferol has been found to possess significant anti-tumour potential *in vitro* and *in vivo*, exhibiting dose-dependent activity against several types of cancer cells, including pancreatic cancer (Wang et al., 2021), lung cancer (Kuo et al., 2015), hepatocellular carcinoma (Yang et al., 2021), and colorectal carcinoma (Wu et al., 2022). Its mechanism of action involves inhibiting tumour cell growth, promoting apoptosis, and preventing tumour cell proliferation (Table 1).

**TABLE 1:** Possible mechanisms, real modules, targets, doses and reference of kaempferol in gynaecological malignant tumours.

Possible mechanisms	Cancer	Real modules (animal/cell)	Targets	Doses	References
Apoptosis	Breast Cancer	MCF-7	PARP, caspase-7, Bax, caspase-9, PLK-1	50 μM	Kang et al. (2009)
		ZR-75–30, BT474	IQGAP3, ERK1/2	10, 25, 50, 100 μM	Hu et al. (2019)
		MCF-7	caspase-9, caspase-3, PARP	NA	Diantini et al. (2012)
		MCF-7	Bcl2	20, 40, 80 μM	Yi et al. (2016)
		38 patients’ Tumor explants	p53, CD44, ALDH1, NANOG, MDR1, Ki67, Bcl-2, Caspase 3	224.51 μM	Nandi et al. (2022a)
		MCF-7	ERK	30 μM	Kim et al. (2008)
	Ovarian cancer	OVCAR-3, SKOV-3	ERK, JNK, CHOP, DR4, DR5, Bcl-xl, Bcl-2, surviving, XIAP, c-FLIP, caspase-3, caspase-8, caspase-9, Bax	20–100 μM	Zhao et al. (2017)
	Cervical cancer	HeLa	PI3K, AKT, hTERT	12–100 μM	Kashafi et al. (2017)
	Endometrial cancer	HEC-265, HEC108, HEC180	ERα、survivin, Bcl-2	36, 72 μM	Chuwa et al. (2018)
Proliferation	Breast Cancer	MCF-7	glut1	30,100 μM	Azevedo et al. (2015)
		MCF-7	Cyclin-D1, cyclin-E, cathepsin, p21, bax, pIRS-1, pAkt, pMEK1/2	50–100 μM	Kim et al. (2016)
	Ovarian cancer	OVCAR-3	Caspase-3, caspase-8, caspase-9, Bax, G2/M, MEK/ERK, STAT3	25–50 μM	Yang et al. (2019)
	Cervical cancer	SiHa	Ca^2+^	40 mg/mL	Tu et al. (2016)
Cell cycle	Breast Cancer	MDA-MB-453	G2/M, CDK1, cyclin A, cyclin B, p53	10, 50 μM	Choi and Ahn (2008)
		MDA-MB-231	G2/M, γH2AX, p-ATM, cleaved caspase-9, cleaved caspase-3, p-ATM	50 μM	Zhu and Xue (2019)
	Ovarian cancer	A2780, CP70	G2/M, Chk2Cdc25C/Cdc2, Chk2/p21/Cdc2	40 μM	Gao et al. (2018)
	Endometrial cancer	MFE-280	G2/M, TORPI3K/AKT	10 μM	Lei et al. (2019)
Invasion and metastasis	Breast Cancer	MDA-MB-231	PKC/MAPK/AP-1, MMP-9	40 μM	Li et al. (2015)
		4T-1	ROS-PAD4, H3-cit	25 μM	Zeng et al. (2020)
		MDA-MB-231, MDA-MB-453, MCF-7, SK-BR-3	RhoA, Rac1	20 μM	Li et al. (2017)
		MCF-7	EMT, N-cadherin、Snail, Slug, Cathepsin B	25 μM	Lee et al. (2017)
	Ovarian cancer	HACAT, AGS, SKOV3IP1, MDA-MB-231	EMT, TGF-β/ALK5/Smad	2 μM	Zhang et al. (2021)
Autophagy	Breast Cancer	MCF-7	CYP19, CYP17a, CCND2, GDF9, INSL3, ER1, ER2	15, 30 μM	Harrath et al. (2021)
Tumour angiogenesis	Ovarian cancer	OVCAR-3	VEGF	20 μM	(Luo et al., 2008)
		OVCAR-3, A2780/CP70	p53	0–80 μM	Luo et al. (2011)
		OVCAR-3, A2780/CP70	ERK/NF-κB/cMyc/p21/VEGF	20 μM	Luo et al. (2012)

### 3.1 Induction of apoptosis in tumour cells

Apoptosis is a mechanism in the cell cycle that maintains a check and balance, eliminating non-functional, harmful, abnormal and misplaced cells in a timely manner (Kaczanowski, 2016). This process is important in preventing tumour development, as one of the defining characteristics of tumour cells is the inhibition of apoptosis (Ucker and Levine, 2018).

Apoptosis pathways are commonly classified into exogenous, endogenous (mitochondrial), and endoplasmic reticulum stress-induced pathways (Pfeffer and Singh, 2018). Kaempferol has been demonstrated to play a significant role in breast cancer apoptosis, according to a number of studies. Kang et al. (2009) conducted a study on the MCF-7 human breast cancer cells, utilizing kaempferol as the induction factor for apoptosis. They were able to activate caspase-9 and downregulate PLK-1 expression, a protein vital for mitotic progression and commonly upregulated in various human tumours. In another study, Hu et al. (2019) posited QGAP3 as a potential target gene for kaempferol treatment of BC. They discovered that upregulated IQGAP3 hindered kaempferol-induced apoptosis in BC cells through activation of ERK1/2 signalling. Diantini et al. (2012) discovered that Kaempferol-3-O-rhamnoside halts cell proliferation in a dose-dependent manner and encourages apoptosis by initiating the caspase signalling process (including caspase-9, caspase-3 and PARP) in MCF-7 breast cancer cells. According to Yi et al. (2016), kaempferol can curb the growth and trigger apoptosis in MCF-7 breast cancer cells by a decrease in Bcl2 expression. Nandi et al. (2022a) demonstrated the potential effectiveness of kaempferol in vitro-grown breast tumours of triple-negative breast cancer patients after NACT by down-regulating nuclear p53, CD44, ALDH1, NANOG, MDR1, Ki67, Bcl-2 and up-regulating Caspase 3. Kim et al. (2008) discovered that under 3-D culture conditions, kaempferol more significantly induces apoptosis in MCF-7 breast cancer cells by activating ERK.

**FIGURE 1:**
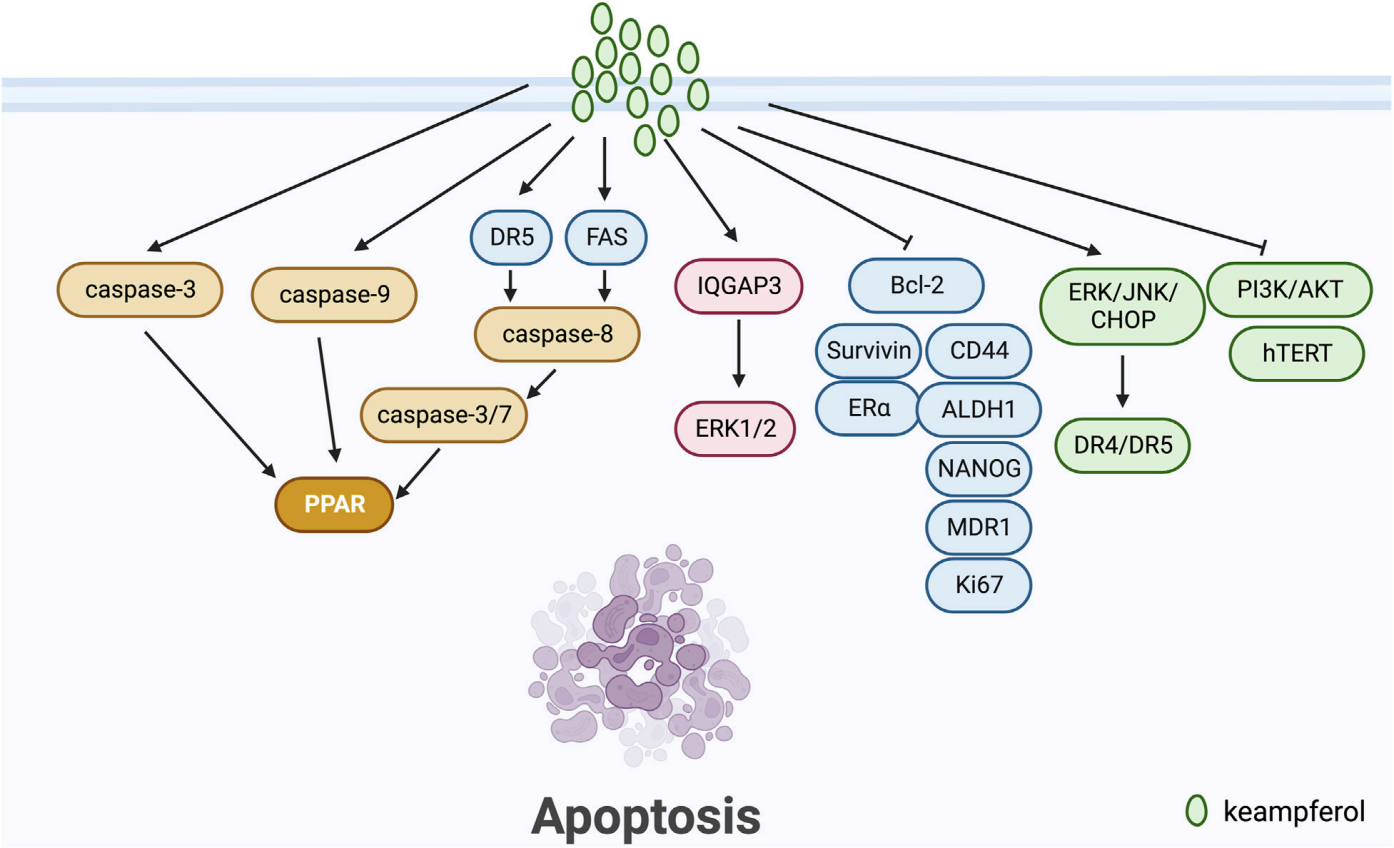
Mechanism of kaempferol inducing apoptosis in gynaecological malignancies.

Kaempferol has been extensively studied for its role in inducing apoptosis in tumour cells of ovarian, cervical, and endometrial cancers. According to Zhao et al. (2017), kaempferol upregulates the expression of DR4 and DR5 through the ERK/JNK/CHOP signalling pathway, resulting in apoptosis in ovarian cancer cells. Kashafi et al. (2017) indicate that kaempferol causes apoptosis in human HeLa cervical cancer cells by inhibiting PI3K/AKT and hTERT in a concentration-dependent and time-dependent manner. Meanwhile, Chuwa et al. (2018) demonstrate that kaempferol stimulates apoptosis in endometrial cancer cells primarily by inhibiting ERα, survivin, and Bcl-2 proteins. This leads to an increase in cancer cell apoptosis (Figure 1).

### 3.2 Inhibition of tumour cell proliferation

Cell proliferation is a key factor in cell growth and differentiation, and inhibiting tumour cell proliferation is crucial in anti-tumour therapy (Patra et al., 2023).

Kaempferol impacts the growth of gynaecological cancer cells by impeding their proliferation. A study by Azevedo et al. (2015) revealed that kaempferol prevented glut1-mediated glucose uptake in MCF-7 breast cancer cell lines, resulting in cytotoxicity and proliferation inhibition. Additionally, the research team reported that kaempferol hindered MCT1, thus impeding lactate reuptake in these cells. Kaempferol inhibits lactate reuptake in breast cancer cells, resulting in a lactate deficiency causing cell death. Kim et al. (2016) discovered its ability to significantly impede TCS and E2-induced cell proliferation in breast cancer, acting as an ER and IGF-1R signalling antagonist while also down-regulating protein expressions of cyclin D1, cyclin E, and cathepsin D, and up-regulating those of p21, Bax, pIRS-1, pAkt, and pMEK1/2. Yang et al. (2019) discovered that kaempferol elicits antiproliferative effects on OVCAR-3 human ovarian cancer cells by upregulating the expression of apoptotic proteins, namely, caspase 3, caspase 8, caspase 9, and Bax, which induce apoptosis, G0/G1 cell-cycle blockade, and modulation of the MEK/ERK and STAT3 pathways. Tu et al. (2016) demonstrated, at a macro-to nano-level, that kaempferol promotes apoptosis and inhibits proliferation in human cervical cancer SiHa cells.

**FIGURE 2:**
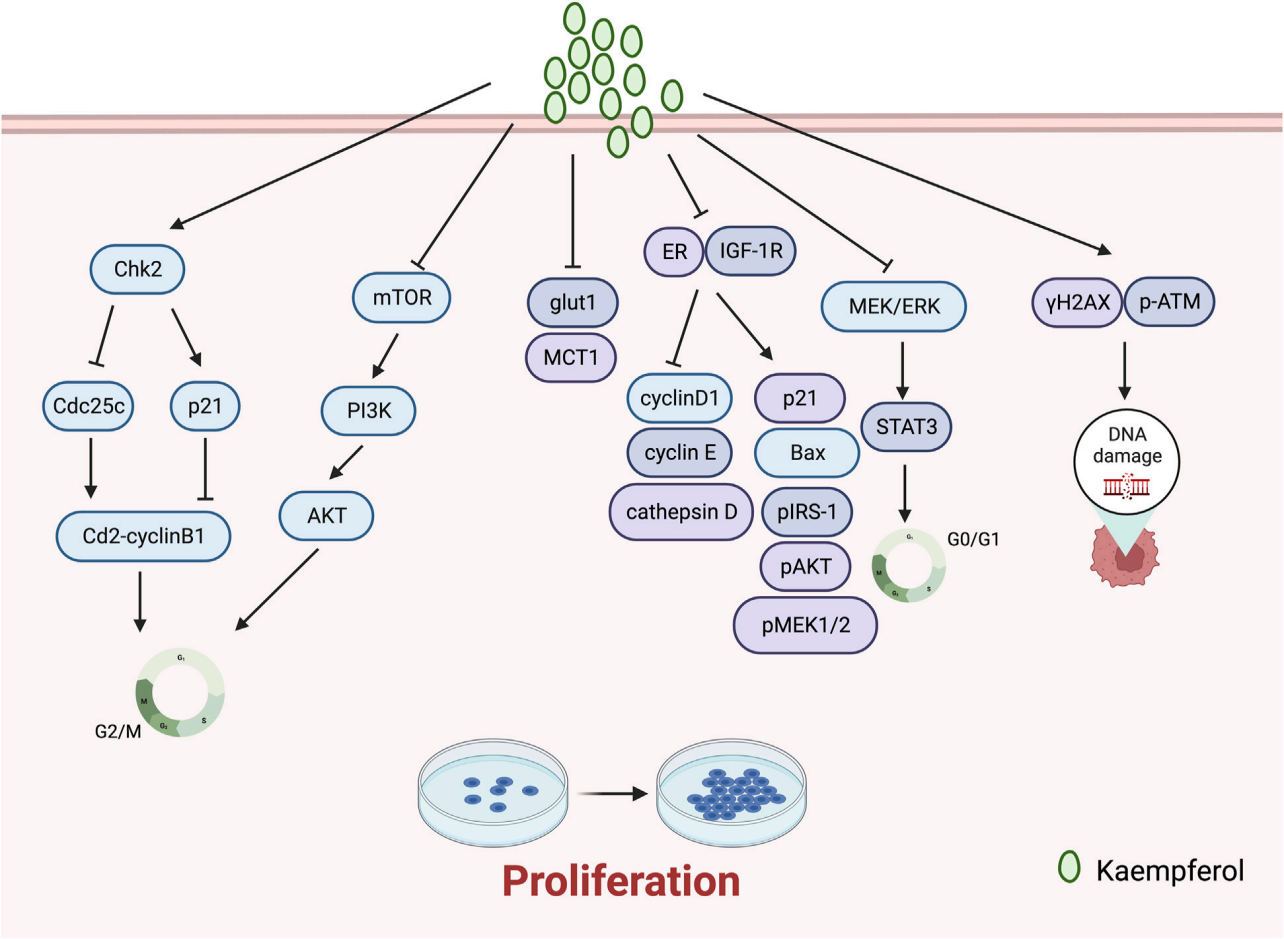
Mechanism of kaempferol suppressing proliferation in gynaecological malignancies.

The literature has reported unexpected therapeutic effects in inhibiting the proliferation of breast cancer through the synergistic use of kaempferol with other drugs. Ackland et al. (2005) discovered that quercetin and kaempferol synergistically reduce cell proliferation in the PMC42 human mammary cell line at physiological concentrations between 1 and 10 of 5 ÌM. Afzal et al. (2023) reported that the combination of kaempferol and laccasein is more effective in inhibiting the proliferation of TNBC cancer cells (MDA-MB-231) than either substance alone. The co-administration of kaempferol and laccasein resulted in the downregulation of p-Akt protein expression and inhibition of the PI3K/Akt pathway. This, in turn, hindered the advancement of breast cancer through the induction of ROS-mediated DNA damage and mitochondria-mediated apoptosis pathways (Figure 2).

### 3.3 Induction of tumour cell cycle block

Cell proliferation resulting from aberrations in the cell cycle is a fundamental trait of neoplastic cells (Kar, 2016). Therefore, hindering the cycle progression of tumour cells becomes an essential method of inhibiting tumour cell proliferation.

G2/M cell cycle arrest is a usual cellular response to DNA damage caused by agents like radiation, and is associated with genomic instability, tumourigenesis, and treatment (Jamasbi et al., 2022). Notably, recent studies have revealed that kaempferol frequently induces cell cycle arrest at the G2/M phase during the treatment of gynaecological malignancies. Kaempferol inhibits the growth of human breast cancer MDA-MB-453 cells by disrupting the cell cycle. It induces cell cycle arrest at the G2/M checkpoint and is thought to promote apoptosis via p53 phosphorylation (Choi and Ahn, 2008). In addition, Zhu and Xue (2019) discovered that treatment with kaempferol induces G2/M phase arrest, apoptosis, and DNA damage in MDA-MB-231 cells. The experimental data indicates that kaempferol augments the expression of γH2AX and p-ATM, leading to DNA damage. Kaempferol constrains the growth of A2780/CP70 human ovarian cancer cells through Chk2 and death receptors (Gao et al., 2018). Specifically, kaempferol causes a blockage in the G2/M phase of human ovarian cancer cells by means of the Chk2/Cdc25C/Cdc2 pathway and the Chk2/p21/Cdc2 pathway. Lei et al. (2019) discovered that kaempferol causes endometrial cancer cell inhibition by stimulating apoptosis, obstructing the G2/M phase cell cycle, hindering cell invasion, and activating the TOR/PI3K/AKT signalling pathway. Nandi et al. (2022b) assessed the effects of combining kaempferol with verapamil, an MDR1 inhibitor, on the growth of breast cancer stem cells. They found that the combination of KV induced G2/M-dependent cell cycle block and disrupted the physical binding of CD44 to NANOG and MDR1. (Figure 2).

### 3.4 Inhibition of tumour cell metastasis and invasion

Tumour cell invasion and metastasis are typical characteristics of malignant tumours. The primary indicators are MMP-2, MMP-9, N-cadherin, and E-cadherin (Yilmaz et al., 2007; Wan et al., 2013). Li et al. (2015) discovered that kaempferol inhibits the invasion of MDA-MB-231 breast cancer cells by blocking the PKC/MAPK/AP-1 pathway and decreasing the MMP-9 expression. Zeng et al. (2020) proposed that kaempferol inhibits the formation of NETs and reduces their occurrence by inhibiting the ROS-PAD4 pathway. Traps formation, H3-cit expression reduction, and breast cancer cell metastasis inhibition were observed in mice. Li et al. (2017) demonstrated that low-dose kaempferol (20 μmol/L) obstructed the RhoA and Rac1 signalling pathways, subsequently restraining the migration and invasion of TNBC cells.

**FIGURE 3:**
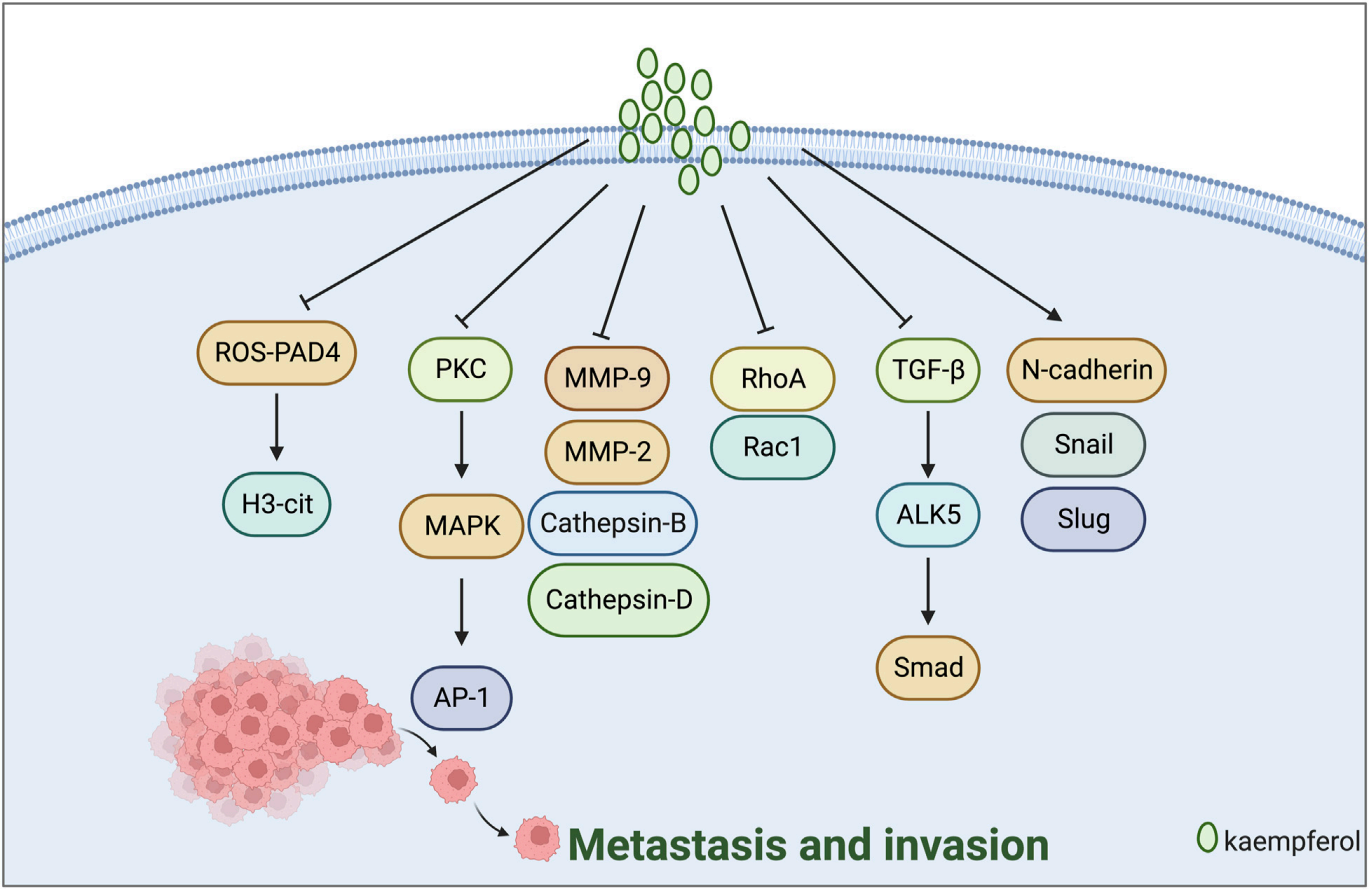
Mechanism of kaempferol suppressing metastasis and invasion in gynaecological malignancies.

EMT refers to the acquisition of mesenchymal cell characteristics by epithelial cells, which contributes to the enhanced invasion and migration of tumour cells. This process is associated with reduced expression of epithelial cell markers such as E-cadherin and increased expression of mesenchymal cell markers like N-cadherin (Mittal, 2018; Dongre and Weinberg, 2019). Lee et al. (2017) discovered that kaempferol inhibits EMT, migration, and invasion of MCF-7 breast cancer cells via ER. This is achieved by regulating the protein expression of genes related to EMT and metastasis, such as N-cadherin, Snail, Slug, and Cathepsin B. Technical abbreviations are explained when first used. Zhang et al. (2021) discovered that Kaempferol 3-O-gentiobioside prevented the migration and invasion of cancer cells, reversed the expression of EMT-related regulatory factors, and reduced tumour growth *in vivo* by blocking the classical TGF-β/ALK5/Smad pathway (Figure 3).

### 3.5 Inducement of tumor cell autophagy

Autophagy is an intracellular process that breaks down impaired proteins or organelles, depositing them in lysosomes for cellular recycling. It is essential for maintaining healthy cellular function (Li et al., 2020; Gao et al., 2022).

**FIGURE 4:**
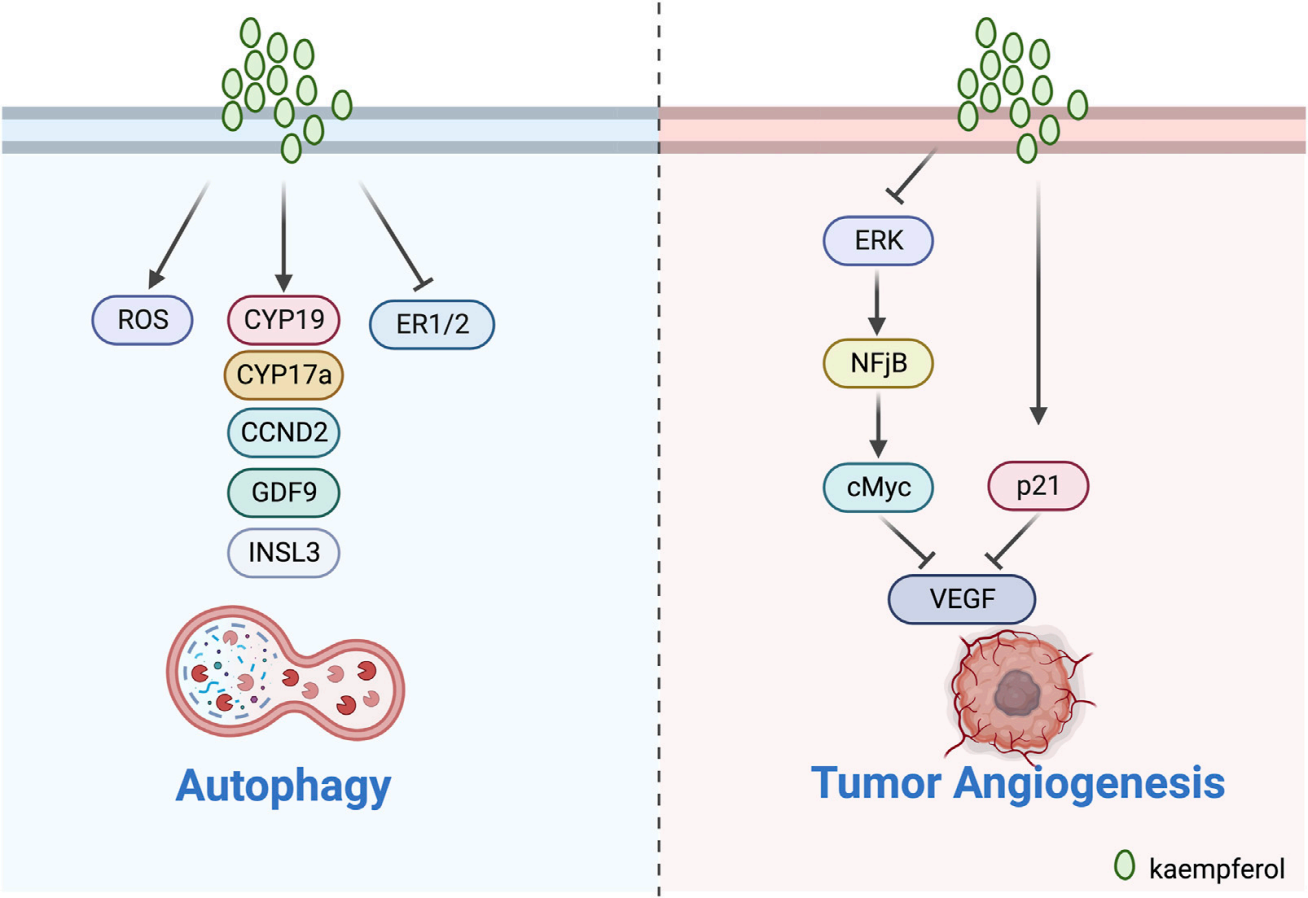
Mechanism of kaempferol inducing autophagy and inhibit tumour angiogenesis in gynaecological malignancies.

Nandi et al. (2023) treated BC cell lines (MDA-MB-231) with a combination of KV. The experimental evidence indicated that KV produced excessive ROS under low glucose conditions. The treatment also downregulated markers of chemo-resistance and tumour acidosis, as well as ATP1B1. This led to lysosomal disruption, reduced Ca2^+^ release and TFEB expression. Furthermore, KV triggers the overproduction of ROS by upregulating LC3-II and p62, leading to autophagy-mediated cell death. Harrath et al. (2021) discovered that kaempferol-3-O-apiofuranosyl-7-O-rhamnopyranosyl can enhance ROS production, inducing dose-dependent autophagy and apoptosis of MCF-7 breast cancer cells. The findings suggest that the compound may be a promising treatment for breast cancer patients (Figure 4).

### 3.6 Inhibition of tumour angiogenesis

Tumour angiogenesis is crucial for tumour growth and metastasis as it requires a blood supply for oxygen and nutrient provision as well as other metabolic processes (Apte et al., 2019; Ahmad and Nawaz, 2022). VEGF is closely associated with tumour angiogenesis.

Luo’s team illustrated the significant function of kaempferol in ovarian cancer by influencing VEGF through several experiments. According to Luo et al. (2008), kaempferol suppressed the proliferation of OVCAR3 ovarian cancer cells in a dose-dependent manner by reducing the expression of VEGF protein. In addition, Luo et al. (2011) also observed that kaempferol modestly limited cell viability through an anti-angiogenic assay in two ovarian cancer cell lines (OVCAR-3 and A2780/CP70). The results of the experiment showed that kaempferol curbed angiogenesis and VEGF expression in human ovarian cancer cells via HIF Dependent and Independent Pathways. Luo et al. (2012) confirmed the effectiveness of kaempferol in lessening the chances of ovarian cancer. Their experimental analysis displayed that kaempferol impeded VEGF secretion in a timely manner via the ERK-NF-κB-cMyc-p21-VEGF pathway and repressed angiogenesis *in vitro*. Kaempferol decreases the relationship between ERK phosphorylation and expression of NF-κB and cMyc, but increases expression of p21 (Figure 4).

### 3.7 Increased antitumour drug sensitivity

Natural plants, including botanicals, contain a plethora of biologically active substances. As research technology advances, natural medicines are increasingly employed in conjunction with antitumour drugs to offer significant benefits to tumour prevention and treatment (Alsanad et al., 2014). Researchers are also becoming increasingly interested in the co-administration of kaempferol in gynaecological oncology (Table 2).

**TABLE 2:** Drug combination, cancer, real modules, possible mechanisms, targets, doses and reference of kaempferol in gynaecological malignant tumours.

Drug combination	Cancer	Real modules	Possible mechanisms	Targets	Doses	References
Kaempferol + cisplatin	Ovarian cancer	OVCAR-3	Apoptosis	ABCC6, cMyc, CDKN1A	80μM + 20 μM	Luo et al. (2010)
Kaempferol + cisplatin		A2780	Autophagy, Cell Death	PI3K/Akt, p53	40 μM + (0–20) μM	El-Kott et al. (2020)
Kaempferol + Verapamil	Breast Cancer	MDA-MB-231	Cell cycle	SOX2, OCT4, NANOG, MDR1, CD44, γ-h2ax	(104.8–109.9) μM + 5 μM	Nandi et al. (2022a)
Kaempferol + Verapamil		MDA-MB-231, MCF-7	Autophagy	LC3-II, p62, TFEB, ATP1B1	104.8μM + 5 μM	Nandi et al. (2023)

The nucleophilic amines present in platinum drugs can react with water molecules, generating free radicals. Such reactions cause harm to cell membranes, mitochondrial membranes, and other biological membranes. The toxicity of these drugs is evidenced by a reduction in the glomerular filtration rate and an accompanying increase in blood urea nitrogen, blood creatinine, N-acetyl-D-amino-glucosidase, proteinuria, polyuria, polydipsia, oliguria, and haematuria (Dasari and Tchounwou, 2014; Ghosh, 2019). In recent years, multiple studies have demonstrated that natural medicines, when used alongside cisplatin, can significantly diminish gastrointestinal responses such as chest and epigastric tightness, anorexia, malaise, vomiting, amongst others, as well as bone marrow suppression and simultaneously reduce toxicity while increasing the effectiveness of treatment (Qian et al., 2010; Al Sawah et al., 2015). According to Luo et al. (2010), kaempferol presents itself as an essential component in synergizing with cisplatin. The researchers’ findings demonstrated that the combination of cisplatin and 20 μM kaempferol resulted in the induction of cancer cell apoptosis. Furthermore, kaempferol amplified the effect of cisplatin by down-regulating ABCC6 and cMyc expression, while up-regulating CDKN1A expression to advance apoptosis in OVCAR-3 human ovarian cancer cells. El-Kott et al. (2020) proposed that kaempferol could serve as a novel chemotherapeutic agent in triggering ovarian carcinoma cell death. Furthermore, it could enhance the potency of chemotherapy by obstructing the PI3K/Akt signalling pathway, thus increasing the sensitivity of ovarian carcinoma cells towards cisplatin.

**FIGURE 5:**
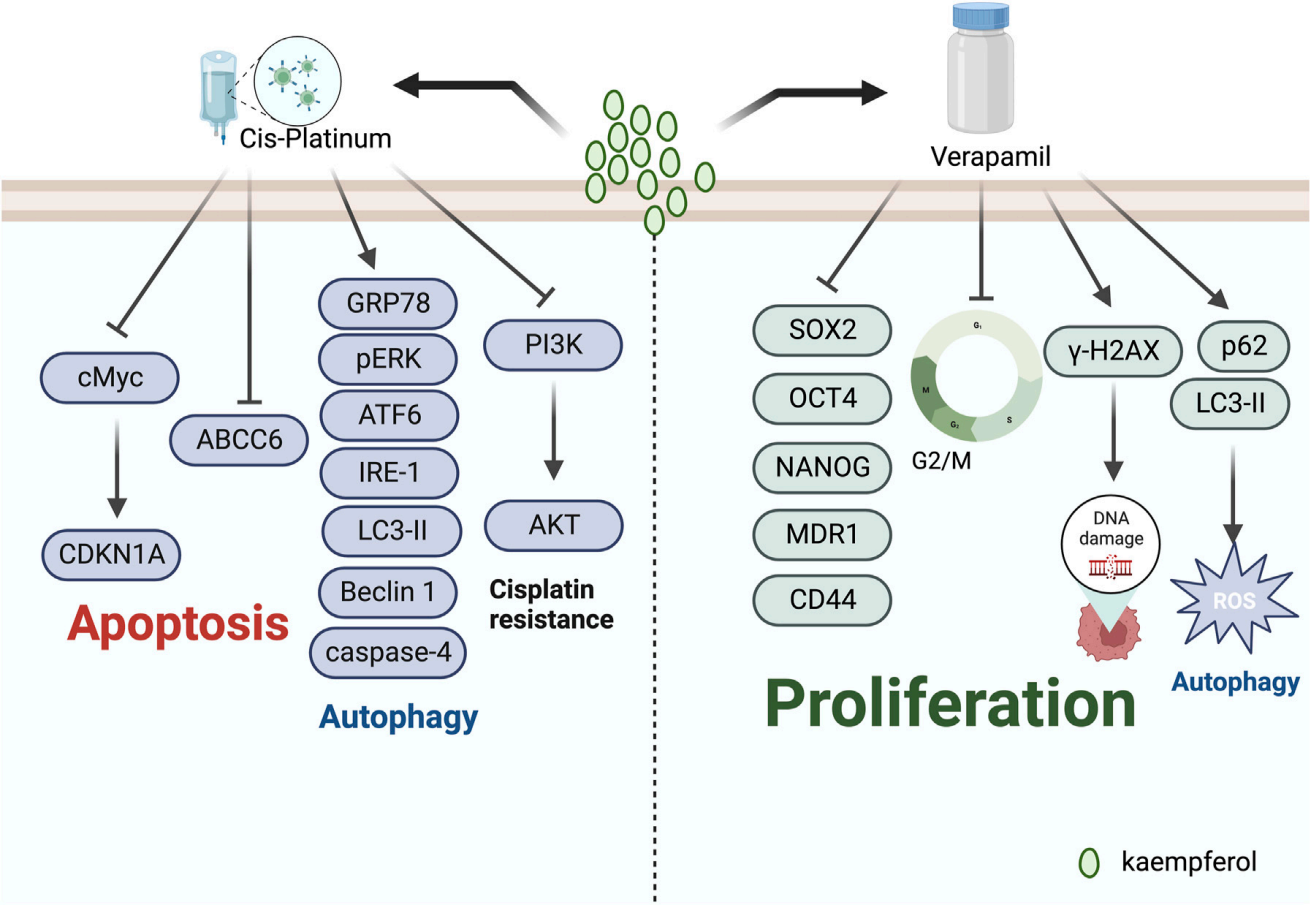
Mechanism of kaempferol inducing antitumour drug sensitivity in gynaecological malignancies.

Verapamil is a racemic compound of phenylalkylamine, which effectively blocks L-type calcium channels, thus hindering the influx of extracellular calcium ions into myocardial and vascular smooth muscle cells, and is widely used for treating hypertension, angina pectoris, and arrhythmias (McTavish and Sorkin, 1989). Additionally, studies have demonstrated that verapamil has the ability to reverse multidrug resistance in tumour cells by inhibiting drug efflux pump proteins (La Vecchia and Bosetti, 2003; Shiozaki et al., 2021). In the treatment of ovarian cancer, verapamil and kaempferol in combination have been suggested as more effective, according to some researchers. Nandi et al. (2022a) conducted a study on breast cancer stem cells, using kaempferol alone and along with the MDR1 inhibitor verapamil. Their research discovered that the anti-stem cell impact of KV co-treatment was stronger, leading to attenuation of SOX2, OCT4, NANOG, MDR1, and CD44 expression and promotion of γ-H2AX expression. Furthermore, the combined use of KV was found to be more effective than using kaempferol alone in the course of treatment. Moreover, Nandi et al. (2023) conducted an intervention with the combination of KV in MDA-MB-231 breast cancer cell lines under conditions of low glucose. Their study indicated that KV reduced chemo-resistance by increasing the expression of LC3-II and p62 proteins, inducing excessive ROS, and activating cellular autophagy. Their study indicated that KV reduced chemo-resistance by increasing the expression of LC3-II and p62 proteins, inducing excessive ROS, and activating cellular autophagy. Their study indicated that KV reduced chemo-resistance by increasing the expression of LC3-II and p62 proteins, inducing excessive ROS, and activating cellular autophagy (Figure 5).

## 4 Discussion and perspectives

In recent years, the incidence and fatality rates of gynaecological malignancies have been steadily increasing, drawing greater attention from scholars. The use of natural remedies is now well-established in this field, as evidenced by their notable effects. Recently, chemical compounds found in these remedies have been isolated and widely employed in clinical settings, where they have demonstrated superior efficacy. Flavonoids are a group of secondary metabolites manufactured by plants through long-term natural selection. Flavonoids are a subclass of polyphenols and were first discovered as substances that are yellow or yellowish, hence the name flavonoids. Flavonoids are presently under investigation for nutritional and pharmaceutical product development. This specific class of compounds, found within the herbal system of traditional Chinese medicine, offers clear advantages for a wide range of applications, such as skin care, inflammation reduction, immunity boosting, and other product formulations. Kaempferol, as a natural flavonoid, may have a wide-ranging potential for anti-tumour effects.

Kaempferol is extracted from various fruits, vegetables and herbs with minimal toxicity and few adverse reactions. Medical practitioners have taken an interest in kaempferol due to its numerous pharmacological effects including its antioxidant, anti-inflammatory and neuroprotective properties. Notably, kaempferol has been observed to possess highly effective anti-tumour potential against malignant tumours such as breast, lung, liver and pancreatic cancers. Several studies have demonstrated that kaempferol has the potential to induce apoptosis of tumour cells, inhibit the proliferation of tumour cells, prevent metastasis and invasion, and induce tumour cell autophagy in gynaecological malignant tumours, including breast cancer, ovarian cancer, and endometrial cancer. The mechanism of its anti-tumour activity can induce apoptosis in tumours as it regulates the PI3K/Akt pathway and ROS. Its anti-proliferative quality positively impacts the PI3K/Akt pathway. Furthermore, it mainly regulates cyclin and CDK, thereby exhibiting antiproliferative activity. Additionally, it is effective in inhibiting the metastasis and invasion of tumour cells by obstructing the EMT process. Furthermore, kaempferol has the potential to increase the efficacy of antitumour drugs and initiate autophagy in tumour cells, contributing to its role in combatting gynaecological malignant tumours. However, research into the effects of kaempferol on gynaecological malignancies remains primarily limited to *in vitro* studies, with fewer *in vivo* studies available. Additionally, studies on kaempferol in relation to breast cancer offer little novel information compared to other types of tumours. Given these gaps in knowledge, further exploration of the role of kaempferol in gynaecological malignancies is warranted in future studies.

Kaempferol has not yet been formulated for clinical use, potentially due to the limited number of *in vivo* and vitro experiments and clinical studies, and the specificity of the compound. The precise way in which kaempferol works and its inhibitory effects on tumours, together with the associated targets in clinical settings, are important aspects that require focused exploration. Furthermore, a significant proportion of natural active ingredients typically exhibit low water solubility and lack robust pharmacological activity. They also possess complex, unclear targets and poor metabolic stability, along with non-specific adsorption defects. Moreover, the impediments to chemical synthesis and limited scope for structural modification significantly hamper the clinical translation of botanical medicine’s active ingredients into new drugs. With advances in science and technology, the anti-cancer properties of kaempferol will be elucidated further and can be utilised more effectively in cancer treatment.

## Figures and Tables

**Table:** 

**Chk2**	Checkpoint kinase 2
**Cdc25c**	cell division cycle 25C Gene
**P21**	RAC1
**mTOR**	mammalian target of rapamycin
**PI3K**	Phosphoinositide 3-Kinase
**AKT**	protein kinase B
**glut1**	Glucose transporter type 1
**MCT1**	Monocarboxylate Transporter 1
**ER**	endoplasmic reticulum
**IGF-1R**	Insulin-like growth factor 1 receptor
**Bax**	BCL2-associated X protein
**pIRS-1**	Phosphorylated - Insulin Receptor Substrate-1
**pAKT**	Phosphorylated protein kinase B
**Pmek1/2**	Phospho-MEK1/2
**MEK**	Mitogen-activated protein kinase kinase
**ERK**	Extracellular regulated protein kinases
**STAT3**	Signal transducer and activator of transcription 3
**γ-H2AX**	gamma-H2AX
**pATM**	Phosphorylated- ataxia telangiectasia-mutated gene
**ROS**	Reactive oxygen species
**CYP19**	aromatase CYP19
**CYP17a**	cytochrome P450 family 17 subfamily A
**CCND2**	cyclin D2
**GDF9**	Growth Differentiation Factor 9
**INSL3**	Insulin-like peptide 3
**ER1/2**	extracellular regulated protein kinases
**NF-κB**	nuclear factor kappa-B
**VEGF**	Vascular endothelial growth factor
**ROS-PAD4**	Peptidyl arginine deiminase 4
**H3-cit**	Citrullinated histone H3
**PCK**	Protein kinase C
**MAPK**	Mitogen-activated protein kinases
**AP-1**	Activator protein-1
**MMP-9**	Matrix metallopeptidase 9
**MMP-2**	Matrix metalloproteinase 2
**RhoA**	Ras homolog gene family, member A
**Rac1**	activated kinase 1 Gene
**TGF-β**	Transforming growth factor beta
**ALK5**	anaplastic lymphoma kinase 5
**Smad**	*drosophila* mothers against decapentaplegic protein
**N-cadherin**	Neural-cadherin
**Snail**	snail family transcriptional repressor
**Slug**	SNAI2
**Caspase-3**	Cysteine-requiring Aspartate Protease3
**Caspase-9**	Cysteine-requiring Aspartate Protease 9
**DR5**	Death receptor 5
**FAS**	TNF superfamily receptor 6
**Caspase-8**	Cysteine-requiring Aspartate Protease 8
**Caspase-3**	Cysteine-requiring Aspartate Protease 3
**PPAR**	Peroxisome proliferator-activated receptor
**IQGAP3**	IQ Motif Containing GTPase Activating Protein 3
**ERK1/2**	Extracellular signal-regulated kinase1/2
**Bcl-2**	B-cell lymphoma-2
**ERα**	estrogen receptor α
**CD44**	homing cell adhesion molecule
**ALDH1**	Aldehyde de-hydrogenase 1
**MDR1**	Multidrug Resistance Protein 1
**Ki67**	MKI67
**JNK**	Jun N-terminal kinase
**CHOP**	C/EBP homologous protein 10
**DR4**	Death receptor 4
**DR5**	Death receptor 5
**hTERT**	Human telomerase reverse transciptase
**CDKN1A**	Cyclin dependent kinase inhibitor 1A
**ABCC6**	ATP binding cassette subfamily C member 6
**GRP78**	Glucose-regulated protein 78
**PERK**	Type I transmembrane ER-resident protein kinase
**ATF6**	Activating transcription factor 6
**IRE-1**	Inositol-requiring enzyme 1
**LC3-II**	MAP1LC3 -2
**Beclin 1**	BECN1
**Caspase-4**	Cysteine-requiring Aspartate Protease 4
**SOX2**	Sry-box transcription factors 2
**OCT4**	Octamer-binding transcription factor
**NANOG**	Nanog homeobox Gene
**MDR1**	Multidrug Resistance Protein 1
**P62**	Sequestosome 1
**KV**	kaempferol and verapamil
**NETs**	neutrophil extracellular traps
**EMT**	Epithelial-mesenchymal transition
**VEGF**	Vascular endothelial growth factor
**NACT**	Neo Adjuvant Chemotherapy
