# Supplementary material for: The role of kaempferol in gynaecological malignancies: progress and perspectives
Source: Front Pharmacol. 2023 Dec 4;14:1310416. doi: 10.3389/fphar.2023.1310416 (PMC10748757; doi:10.3389/fphar.2023.1310416)
Supplement: Supplementary file 1 [file Table1.DOCX]

Table 2

Drug combination,cancer, real modules, possible mechanisms, targets, doses and reference of kaempferol in gynaecological malignant tumours.

| Drug combination | Cancer | Real modules | Possible mechanisms | Targets | Doses | Reference |
| --- | --- | --- | --- | --- | --- | --- |
| Kaempferol + cisplatin | Ovarian cancer | OVCAR-3 | Apoptosis | ABCC6, cMyc, CDKN1A | 80 μM + 20 μM | (Luo et al., 2010) |
| Kaempferol + cisplatin |  | A2780 | Autophagy, Cell Death | PI3K/Akt, p53 | 40 μM + (0-20) μM | (El-Kott et al., 2020) |
| Kaempferol + Verapamil | Breast Cancer | MDA-MB-231 | Cell cycle | SOX2, OCT4, NANOG, MDR1, CD44, γ-h2ax | (104.8-109.9) μM + 5 μM | (Nandi, Pradhan, et al., 2022) |
| Kaempferol + Verapamil |  | MDA-MB-231, MCF-7 | Autophagy | LC3-II, p62, TFEB, ATP1B1 | 104.8 μM + 5 μM | (Nandi et al., 2023) |
