# Supplementary material for: The role of kaempferol in gynaecological malignancies: progress and perspectives
Source: Front Pharmacol. 2023 Dec 4;14:1310416. doi: 10.3389/fphar.2023.1310416 (PMC10748757; doi:10.3389/fphar.2023.1310416)
Supplement: Supplementary file 2 [file Table2.DOCX]

Table.1 Possible mechanisms, real modules, targets, doses and reference of kaempferol in gynaecological malignant tumours.

| Possible  mechanisms | Cancer | Real  modules  (animal/  cell) | Targets | Doses | Reference |
| --- | --- | --- | --- | --- | --- |
| Apoptosis | Breast Cancer | MCF-7 | PARP, caspase-7, Bax, caspase-9, PLK-1 | 50 μM | (Kang et al., 2009) |
|  |  | ZR-75-30, BT474 | IQGAP3, ERK1/2 | 10, 25, 50, 100 μM | (Hu et al., 2019) |
|  |  | MCF-7 | caspase-9, caspase-3, PARP | NA | (Diantini et al., 2012) |
|  |  | MCF-7 | Bcl2 | 20, 40, 80 μM | (Yi et al., 2016) |
|  |  | 38 patients’ Tumor explants | p53, CD44, ALDH1, NANOG, MDR1, Ki67, Bcl-2, Caspase 3 | 224.51 μM | (Nandi, Pradhan, et al., 2022) |
|  |  | MCF-7 | ERK | 30 μM | (Kim et al., 2008) |
|  | Ovarian cancer | OVCAR-3, SKOV-3 | ERK, JNK, CHOP, DR4, DR5, Bcl-xl, Bcl-2, surviving, XIAP, c-FLIP, caspase-3, caspase-8, caspase-9, Bax | 20 ~ 100 μM | (Zhao et al., 2017) |
|  | Cervical cancer | HeLa | PI3K, AKT, hTERT | 12 ~ 100 μM | (Kashafi et al., 2017) |
|  | Endometrial cancer | HEC‑265, HEC108, HEC180 | ERα、survivin, Bcl‑2 | 36, 72 μM | (Chuwa et al., 2018) |
| Proliferation | Breast Cancer | MCF-7 | glut1 | 30,100 μM | (Azevedo et al., 2015) |
|  |  | MCF-7 | Cyclin-D1, cyclin-E, cathepsin, p21, bax, pIRS-1, pAkt, pMEK1/2 | 50 ~ 100 μM | (Kim et al., 2016) |
|  | Ovarian cancer | OVCAR-3 | Caspase-3, caspase-8, caspase-9, Bax, G2/M, MEK/ERK, STAT3 | 25 ~ 50 μM | (Yang et al., 2019) |
|  | Cervical cancer | SiHa | Ca^2+^ | 40 mg/mL | (Tu et al., 2016) |
| Cell cycle | Breast Cancer | MDA-MB-453 | G2/M, CDK1, cyclin A, cyclin B, p53 | 10, 50 μM | (Choi & Ahn, 2008) |
|  |  | MDA-MB-231 | G2/M, γH2AX, p-ATM, cleaved caspase-9, cleaved caspase-3, p-ATM | 50 μM | (Zhu & Xue, 2019) |
|  | Ovarian cancer | A2780, CP70 | G2/M, Chk2Cdc25C/Cdc2, Chk2/p21/Cdc2 | 40 μM | (Gao et al., 2018) |
|  | Endometrial cancer | MFE-280 | G2/M, TORPI3K/AKT | 10 μM | (Lei et al., 2019) |
| Invasion and metastasis | Breast Cancer | MDA-MB-231 | PKC/MAPK/AP-1, MMP-9 | 40 μM | (Li et al., 2015) |
|  |  | 4T-1 | ROS-PAD4, H3-cit | 25 μM | (Zeng et al., 2020) |
|  |  | MDA-MB-231, MDA-MB-453, MCF-7, SK-BR-3 | RhoA, Rac1 | 20 μM | (Li et al., 2017) |
|  |  | MCF-7 | EMT, N-cadherin、Snail, Slug, Cathepsin B | 25 μM | (Lee et al., 2017) |
|  | Ovarian cancer | HACAT, AGS, SKOV3IP1, MDA-MB-231 | EMT, TGF-β/ALK5/Smad | 2 μM | (Zhang et al., 2021) |
| Autophagy | Breast Cancer | MCF-7 | CYP19, CYP17a, CCND2, GDF9, INSL3, ER1, ER2 | 15, 30 μM | (Harrath et al., 2021) |
| Tumour angiogenesis | Ovarian cancer | OVCAR-3 | VEGF | 20 μM | (Luo et al., 2008), |
|  |  | OVCAR-3, A2780/CP70 | p53 | 0 ~ 80 μM | (Luo et al., 2011) |
|  |  | OVCAR-3, A2780/CP70 | ERK/NF-κB/cMyc/p21/VEGF | 20 μM | (Luo et al., 2012) |
